# Growth signaling promotes chronological aging in budding yeast by inducing superoxide anions that inhibit quiescence

**DOI:** 10.18632/aging.100215

**Published:** 2010-10-27

**Authors:** Martin Weinberger, Ana Mesquita, Timothy Carroll, Laura Marks, Hui Yang, Zhaojie Zhang, Paula Ludovico, William C. Burhans

**Affiliations:** ^1^ Department of Molecular and Cellular Biology, Roswell Park Cancer Institute, Buffalo, NY 14263, USA; ^2^ Instituto de Investigação em Ciências da Vida e Saúde (ICVS), Escola de Ciências da Saúde, Universidade do Minho, Campus de Gualtar, 4710-057 Braga, Portugal; ^3^ Department of Zoology and Physiology, University of Wyoming, Laramie, WY 82071, USA

**Keywords:** oxidative stress, replication stress, lifespan, caloric restriction, hydrogen peroxide

## Abstract

Inhibition of growth signaling pathways protects against aging and age-related diseases in parallel with reduced oxidative stress. The relationships between growth signaling, oxidative stress and aging remain unclear. Here we report that in *Saccharomyces cerevisiae*, alterations in growth signaling pathways impact levels of superoxide anions that promote chronological aging and inhibit growth arrest of stationary phase cells in G0/G1. Factors that decrease intracellular superoxide anions in parallel with enhanced longevity and more efficient G0/G1 arrest include genetic inactivation of growth signaling pathways that inhibit Rim15p, which activates oxidative stress responses, and downregulation of these pathways by caloric restriction. Caloric restriction also reduces superoxide anions independently of Rim15p by elevating levels of H_2_O_2_, which activates superoxide dismutases. In contrast, high glucose or mutations that activate growth signaling accelerate chronological aging in parallel with increased superoxide anions and reduced efficiency of stationary phase G0/G1 arrest. High glucose also activates DNA damage responses and preferentially kills stationary phase cells that fail to arrest growth in G0/G1. These findings suggest that growth signaling promotes chronological aging in budding yeast by elevating superoxide anions that inhibit quiescence and induce DNA replication stress. A similar mechanism likely contributes to aging and age-related diseases in complex eukaryotes.

## INTRODUCTION

Inhibition of growth signaling by caloric restriction (CR) or mutational inactivation of conserved insulin/IGF-1-like and Target of Rapamycin (TOR) signaling pathways prolongs the lifespans of eukaryotic organisms as diverse as yeasts and humans. In complex eukaryotes, inhibition of growth signaling also protects against age-related diseases, including cancer, cardiovascular disease and neurodegenerative disorders [1]. The lifespan-extending effects of reduced growth signaling occur in parallel with the induction of oxidative stress responses that reduce levels of reactive oxygen species (ROS) and oxidative damage to macromolecules. This is consistent with the longstanding free radical theory of aging, which posits oxidative damage to macromolecules as a primary determinant of lifespan [2].

A number of recent studies have investigated the impact of conserved growth signaling pathways on chronological lifespan (CLS) in the model organism *Saccharomyces cerevisiae* (budding yeast). CLS of this organism is determined by measuring the length of time cells maintain viability or reproductive capacity after nutrient depletion induces a stationary phase growth arrest. Growth arrest in stationary phase mimics the quiescent, postmitotic state that occurs in higher eukaryotes when growth-signaling pathways are downregulated during differentiation. The results of many of these studies support the free radical theory. For example, CLS is extended by inactivation of the budding yeast *SCH9* gene encoding a homologue of the mammalian AKT protein [3]. Stationary phase *sch9Δ* cells express elevated levels of the Mn-dependent superoxide dismutase Sod2p [4] and exhibit lower levels of superoxide anions (O_2_**^-^**) [5]. CLS is also extended by inactivation of the mammalian Ras homologue *RAS2* via a mechanism that depends on Sod2p [4] and by inactivation of conserved TOR growth signaling pathways, which also leads to a reduction in levels of O_2_**^-^** [5,6]. When active, Tor1p, Sch9p and Ras2p inhibit the Rim15p kinase and its induction of superoxide dismutases and other oxidative stress defenses, and reduced signaling through these pathways in calorie-restricted cells extends CLS in a Rim15p-dependent fashion [7]. CR or inactivation of catalases also extends CLS by inducing elevated levels of hydrogen peroxide (H_2_O_2_), which inhibits the accumulation of intracellular O_2_**^-^** by activating superoxide dismutases [8]. Conversely, a shorter CLS in concert with elevated levels of O_2_**^-^** have been detected in stationary phase budding yeast cells in which the cAMP phosphodiesterase Pde2p has been inactivated [9]. Pde2 inhibits Ras2p-dependent growth signaling, which is constitutively active in *pde2Δ* cells [10].

All of these findings are consistent with a role for oxidative stress produced in association with growth signaling through highly conserved AKT, TOR and RAS-dependent pathways as an important pro-aging factor in the budding yeast chronological aging model. Similar connections have been established between levels of ROS and growth signaling through AKT, mTOR and RAS-dependent pathways in more complex eukaryotes [11]. However, the mechanisms by which oxidative stress is increased by growth signaling and promotes aging remain unclear. For example, although some of the CLS-extending effects of CR in budding yeast depend on the induction of oxidative and other stress defenses by Rim15p, CR also extends CLS via an Sch9p, Ras2p and Rim15p-independent mechanism [7]. Furthermore, it was recently reported that chronological aging is caused by toxic effects of acetic acid that accumulate in the medium of stationary phase cultures. According to this model, CR or deletion of *SCH9* or *RAS2* extend CLS independently of effects on oxidative stress by inhibiting the accumulation of acetic acid (in the case of CR) or by creating resistance to acetic acid toxicity via unknown mechanisms (in the case of the deletion of *SCH9* or *RAS2*) [12]. In addition, inactivation of catalases extends CLS in parallel with increased levels of oxidative damage to proteins and other macromolecules despite a reduction in intracellular O_2_**^-^** [8]. Although this latter finding is consistent with the model that O_2_**^-^** shortens CLS, it is not consistent with the free radical theory postulate that oxidative damage to macromolecules is a primary cause of aging.

DNA replication stress - i.e., inefficient DNA replication that causes DNA damage - in stationary phase budding yeast cells that fail to arrest in G0/G1 is an additional factor determining CLS of budding yeast. Growth arrest in G0/G1 prevents replication stress that arises when stationary phase cells arrest growth in S phase instead [13]. Experimental manipulations that shorten CLS and inhibit stationary phase growth arrest in G0/G1 include constitutive activation of Ras2p [13,14] and mutational inactivation of Rim15p [15]. In contrast, CR or mutational inactivation of Ras2p or Sch9p promote a more frequent G0/G1 arrest of stationary phase budding yeast cells in concert with an extended CLS [13]. CR was also recently shown to extend the CLS of fission yeast (*S. pombe*) in association with more frequent growth arrest in G0/G1 [16].

To better understand the effects of growth signaling and CR on aging and how they relate to both oxidative and replication stress, in this study we examined levels of ROS in budding yeast stationary phase cells under a variety of experimental conditions in parallel with measurements of CLS and the frequency with which stationary phase cells growth arrest in G0/G1. Our findings indicate that in general, growth signaling enhances the age-dependent accumulation of O_2_**^-^**, and inhibition of growth signaling inhibits O_2_**^-^** accumulation. Furthermore, the reduction in O_2_**^-^** induced by elevated H_2_O_2_ in calorie-restricted cells occurs independently of Rim15p. Although CR inhibits the accumulation of acetic acid in stationary phase cultures under some conditions, it can also reduce intracellular levels of O_2_**^-^** and extend CLS in the absence of changes in levels of acetic acid. Factors that enhance the accumulation of O_2_**^-^** independently of acetic acid include elevated concentrations of glucose, which block the accumulation of H_2_O_2_ and selectively kill cells that fail to arrest in G0/G1 in stationary phase. Together, these findings suggest that growth signaling promotes aging in the chronological aging model in part by inducing O_2_**^-^** that inhibit the growth arrest of stationary phase cells in G0/G1, thus leading to DNA replication stress.

## RESULTS

### Caloric restriction or inactivation of growth signaling pathways reduces superoxide anions in stationary phase cells and enhances G0/G1 arrest

**Figure 1.**
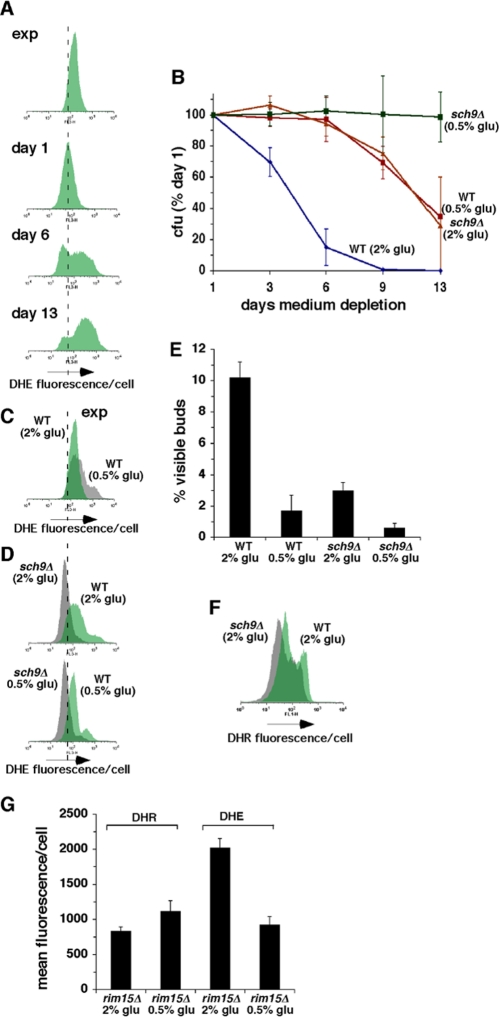
Inhibition of growth signaling pathways prolongs CLS in concert with reduced O_2_^-^ and more frequent growth of stationary phase cells in G0/G1. (**A**) Levels of O_2_**^-^** detected by dihydroethidium (DHE) fluorescence in exponential cultures and stationary phase wild type cells. In this and subsequent figures, dashed vertical line through flow cytometry histograms provides an arbitrarily chosen reference point for comparing related histograms. (**B**) Effects of caloric restriction and/or inactivation of Sch9p on CLS. (**C**) Effect of caloric restriction on levels of O_2_**^-^** detected by DHE fluorescence in exponential cultures of wild type cells. (**D**) Effects of caloric restriction and/or inactivation of Sch9p on levels of O_2_**^-^** detected by DHE fluorescence in stationary phase cells at day 3 of medium depletion. (**E**) Effects of caloric restriction and/or inactivation of Sch9p on the fraction of stationary phase cells that failed to arrest in G0/G1 as measured by counting cells with visible buds at day 3 of medium depletion. (**F**) Levels of H_2_O_2_ detected in stationary phase wild type and *sch9Δ* cells by dihydrorhodamine 123 (DHR) fluorescence at day 3. (**G**) Levels of H_2_O_2_ detected by DHR fluorescence and O_2_**^-^** detected by DHE fluorescence in *rim15Δ* cells at day 3.

Chronological lifespan experiments require the establishment of exponentially dividing cultures of cells that eventually deplete nutrients from the medium, leading to entry into a non-dividing stationary phase state a few days later. Compared to exponential cultures, intra-cellular levels of O_2_**^-^** detected by the fluorescent probe dihydroethidium (DHE), which can detect superoxide [17], initially declined during the few days of experiments but then gradually accumulated with time in stationary phase (Figure 1A). The chronological age-dependent accumulation of O_2_**^-^** occurred in parallel with loss of reproductive capacity as measured by colony-forming units (Figure 1B; “WT 2% glu”). As reported earlier [18], both the accumulation of O_2_**^-^** and loss of reproductive capacity in stationary phase cells were accompanied by an increase in cell death as measured by uptake of the membrane-impermeable DNA stain propidium iodide (PI) (Figure S1), which does not stain viable cells with intact membranes. The rate of cell death was accelerated in cells with visible buds that failed to exit the cell cycle in stationary phase compared to cells without visible buds (Figure S1). This is consistent with an earlier report that a subpopulation of stationary phase cells that includes all budded cells exhibits elevated levels of apoptosis [19].

Also similar to earlier reports [13,20], CR by reducing the initial concentration of glucose in growth medium from 2% to 0.5% extended the CLS of wild type cells (Figure 1B). Although CR initially increased O_2_**^-^** in exponential cultures at the start of CLS experiments (Figure 1C), it led to a reduction in O_2_**^-^** in stationary phase cultures three days later (Figure 1D; compare “WT 0.5% glu” with “WT 2% glu”). This decrease was detected in association with a decrease in the fraction of cells that failed to arrest in G0/G1 as indicated by visible buds (Figure 1E). In the absence of CR, CLS was lengthened to a similar extent (compared to CR) in a strain from which the *SCH9* gene had been deleted, and CR extended the CLS of *sch9Δ* cells even further (Figure 1B). Similar to the effects of CR, the CLS-extending effects of inactivating Sch9p in 2% glucose medium were also accompanied by a decrease in O_2_**^-^**, and inactivation of Sch9p decreased levels of O_2_**^-^** in calorie-restricted cells even further (Figure 1D). A reduction in intracellular O_2_**^-^** in *sch9Δ* cells was recently reported by others as well [5] and is consistent with a previous report that expression of the superoxide dismutase Sod2p is elevated in *sch9Δ* cells [4]. Inactivation of Sch9p also led to a decrease in the fraction of stationary phase cells that failed to arrest growth in G0/G1 as was reported earlier [13], and this fraction was reduced further by CR of *sch9Δ* cells (Figure 1E). Similar quantitative effects on efficiency of G0/G1 arrest in stationary phase in parallel with changes in CLS and O_2_**^-^** were observed in medium containing 2% glucose or 0.5% glucose in cells from which *TOR1* had been deleted (Figure S2). Culturing cells in rich medium (YPD) rather than defined medium (SC), which also promotes more frequent growth arrest of stationary phase cells in G0/G1 [13], also reduced levels of O_2_**^-^** compared to cells cultured in SC (Figure S3).

Growth signaling pathways that depend on Sch9p, Tor1p and Ras2p converge on inhibition of Rim15p kinase activity that activates Sod2p and other stress responses, which are induced when these growth signaling pathways are genetically ablated or attenuated by CR [21]. Sod1p and Sod2p activity are also induced by H_2_O_2_ that accumulates to higher levels in calorie-restricted cells or when catalases have been inactivated [8]. Intracellular levels of H_2_O_2_ detected with the fluorescent probe dihydrorhodamine 123 were slightly reduced in stationary phase *sch9Δ* compared to wild type cells (Figure 1F; “DHR”). Therefore, unlike CR, inactivation of Sch9 does not reduce levels of O_2_**^-^** by inducing elevated levels of H_2_O_2_ that activate SODs. Moreover, CR increased H_2_O_2_ and reduced O_2_**^-^** levels in stationary phase *rim15Δ* cells (Figure 1G), similar to its effects in wild type cells [8]. Thus, the reduction in O_2_**^-^** levels detected in calorie-restricted cells reflects both Rim15p-dependent effects downstream of reduced signaling by Sch9p, Tor1p and Ras2p that do not depend on increased H_2_O_2_, as well as Rim15p-independent effects related to H_2_O_2_ induction of SODs. Rim15p-independent effects of CR on levels of O_2_**^-^** are consistent with an earlier report of Rim15p-independent effects of CR on CLS [7]. Together, these findings reveal the existence of a quantitative relationship between CLS extension, reduction in intracellular levels of O_2_**^-^** by Rim15p-dependent and -independent mechanisms and more efficient growth arrest of stationary phase cells in G0/G1 when growth signaling is inhibited.

### Constitutive activation of growth signaling elevates superoxide anions and inhibits stationary phase G0/G1 arrest

**Figure 2.**
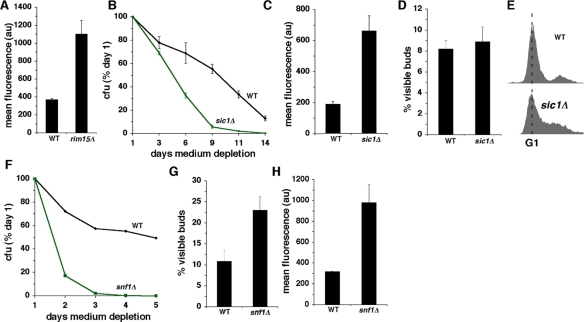
Constitutive activation of growth signaling pathways shortens CLS in concert with increased O_2_^-^ and less frequent growth of stationary phase cells in G0/G1. (**A**) Levels of O_2_**^-^** detected by DHE fluorescence in wild type and *rim15Δ* cells at day 3 of medium depletion. (**B**) CLS of wild type and *sic1Δ* cells. (**C-D**) Levels of DHE fluorescence (**C**) and fraction of cells with visible buds (**D**) in wild type and *sic1Δ* cells. Measurements depicted in panels **C** and **D** were made at day 3 of medium depletion. **E.** DNA content of wild type and *sic1Δ* cells at day 3 of medium depletion. (**F-H**) Effects of inactivating Snf1p on CLS (**F**), fraction of cells with visible buds (**G**) and levels of O_2_**^-^** detected by DHE fluorescence. (**H**) Measurements depicted in panels **G** and **H** were made at day 3 of medium depletion.

In mammals, sustained mitogenic signaling by RAS, AKT and other oncogenes increases levels of O_2_**^-^** and other forms of ROS [22] and induces replication stress in cells that growth arrest in S phase instead of G0/G1 during differentiation [23]. Similar increases in O_2_**^-^** [9] and a reduced frequency of stationary phase growth arrest in G0/G1 phase [13,14] have been described for budding yeast cells expressing constitutively active Ras2p. As noted above, RAS- and AKT-related path-ways that signal growth in response to nutrients in budding yeast converge on inhibition of Rim15p kinase activity [21]. In addition to exhibiting a shorter CLS, *rim15Δ* cells fail to arrest in G0/G1 when they enter stationary phase [13,24]. Stationary phase *rim15Δ* cells also exhibit higher levels of O_2_**^-^** compared to wild type cells (Figure 2A).

The mammalian cyclin-dependent kinase inhibitor p27 blocks entry into S phase when mitogenic growth signaling is downregulated in mammalian cells [25]. Sic1p, the budding yeast homologue of p27, similarly inhibits entry of budding yeast cells into S phase when they enter into a nutrient depletion-induced stationary phase growth arrest [26]. As reported earlier [27], inactivation of Sic1p shortens CLS (Figure 2B). Similar to the effects of the constitutively active Ras2 or deletion of *RIM15*, the shorter CLS of *sic1Δ* cells is accompanied by increased O_2_**^-^** (Figure 2C). Although in this strain background (W303), deletion of *SIC1* did not increase the number of cells with visible buds (Figure 2D), budding is uncoupled from DNA replication in *sic1Δ* cells in some genetic backgrounds [26]. Measurements of DNA content by flow cytometry confirmed that a large fraction of stationary phase W303 *sic1Δ* cells were growth-arrested in S phase, despite a low frequency of buds (Figure 2E). Uncoupling of budding from DNA replication was not observed, however, in *sic1Δ* cells in a different genetic background (CEN.PK). *sic1Δ* cells in this background arrested growth in stationary phase with a substantial increase in the fraction of cells with visible buds (Figure S4).

Snf1p is a conserved AMP kinase that regulates budding yeast metabolism in response to glucose [28]. In mammals, AMPK inhibits mTOR signaling [28] and is required for a “metabolic checkpoint” that drives cells into G1 in response to reduced glucose concentrations [29], similar to the more frequent stationary phase growth arrest in G0/G1 imposed by CR during nutrient depletion of budding yeast cells [13]. In addition to exhibiting a shorter CLS compared to wild type cells (Figure 2F), stationary phase *snf1Δ* cells also arrested in G0/G1 less frequently (Figure 2G) and exhibited elevated levels of O_2_**^-^** (Figure 2H). These findings establish a strong correlation between enhanced growth signaling, increased intracellular levels of O_2_**^-^** and less efficient G0/G1 arrest in stationary phase related to glucose metabolism.

### Enhanced growth signaling by high glucose shortens CLS in parallel with increased superoxide anions, less efficient G0/G1 arrest and increased DNA damage in stationary phase cells

**Figure 3.**
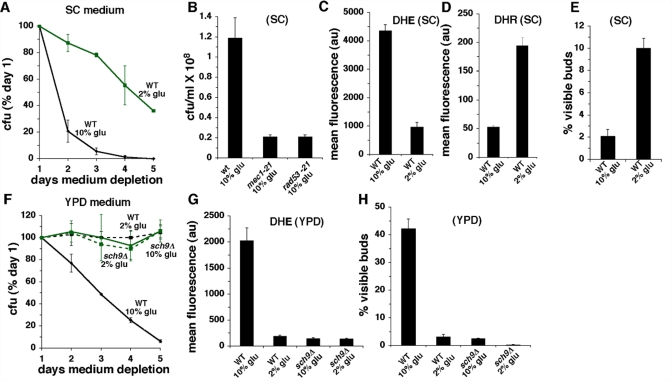
Effects of high glucose (10%) in SC or YPD medium. (**A**) CLS of wild type cells in SC medium initially containing 2% or 10% glucose. (**B**) Reproductive capacity after 2 days of medium depletion of wild type, *mec1-21* or *rad53-21* cells cultured in SC medium containing 10% glucose. (**C**) Levels of O_2_- detected by DHE fluorescence in SC cultures of wild type cells at day 3. (**D**) Levels of H_2_O_2_ detected by DHR fluorescence in SC cultures at day 3. (**E**) Fraction of cells with visible buds in SC cultures at day 3 of medium depletion. (**F**) CLS of wild type and *sch9Δ* cells in YPD medium initially containing 2% or 10% glucose. (**G**) Levels of O_2_- detected by DHE fluorescence in YPD cultures at day 3. (**H**) Fraction of cells with visible buds in YPD cultures at day 3.

High glucose accelerates aging in *C. elegans* [30] and hyperglycemia and/or excess calorie intake are risk factors for a number of age-related diseases. High glucose activates AKT in mammalian cells [31], and similar to enhanced mitogenic signaling by oncogenes [32,33], increased growth signaling by elevated levels of glucose promotes senescence in parallel with DNA damage and increased ROS [34,35]. To determine whether growth signaling by high glucose might trigger related events and accelerate chronological aging in budding yeast, we examined the effects of increasing the concentration of glucose in medium to 10% from the standard 2% (in these experiments, 2% glucose medium also contained 8% sorbitol, a non-metabolized sugar, in order to maintain equivalent osmolarity). Culturing cells in SC medium containing 10% glucose shortened CLS compared to CLS in medium containing 2% glucose (Figure 3A). The shorter CLS of 10% glucose SC cultures is likely accompanied by DNA damage and/or DNA replication stress, because loss of reproductive capacity was dramatically reduced in cells harboring mutations in the DNA damage/DNA replication stress response proteins Mec1p or Rad53p (Figure 3B). Cells cultured in 10% glucose SC medium also exhibited increased levels of intracellular O_2_**^-^** (Figure 3C). This increase occurred in parallel with a reduction in intracellular levels of H_2_O_2_ in 10% glucose compared to 2% glucose cultures (Figure 3D). Since levels of H_2_O_2_are also increased in 0.5% compared to 2% glucose cultures [8], this suggests that glucose inhibits the accumulation of H_2_O_2_ in stationary phase cells in a dose-dependent fashion. The shorter CLS and increased O_2_**^-^** detected in 10% compared to 2% glucose SC cultures was not related to increased medium acidity, because in the genetic background of the strains employed in these experiments (DBY746), the pH of stationary phase cultures established in 10% glucose was not significantly different from the pH of 2% glucose cultures (Table 1). Unexpectedly, the fraction of stationary phase cells with visible buds in 10% glucose SC cultures was reduced rather than increased compared to 2% glucose SC cultures (Figure 3E). This likely reflects the accelerated death of dividing cells in SC medium containing 10% glucose (see below).

**Table 1.** pH day 5 medium depletion

Strain	SC 10% glucose	SC 2% glucose	YPD 10% glucose	YPD 2% glucose
DBY746	3.32 (±0.2)	3.38 (±0.03)	4.72 (±0.11)	4.79 (±0.09)
BY4742	3.19 (±0.02)	3.79 (±0.59)		
BY4741	3.18 (±0.06)	4.17 (±0.07)		
W303	3.17 (±0.01)	3.57 (±0.01)		

Similar to the effects of elevated glucose in SC medium, wild type cells cultured in 10% glucose YPD medium exhibited a shorter CLS and increased O_2_**^-^**levels compared to cells cultured in 2% glucose YPD (Figure 3F-G). However, in contrast to the reduced fraction of cells with visible buds detected in 10% glucose SC (Figure 3E), a substantial increase in the fraction of visibly budded cells was detected in YPD cultures established in 10% glucose (Figure 3H). As was the case for 10% glucose SC medium, the effects of 10% glucose in YPD medium were unrelated to changes in medium acidity, because the pH of these cultures did not differ significantly from the pH of YPD cultures established in 2% glucose (Table 1). Similar to the effects of inactivating Sch9p in cells cultured in 2% glucose SC (Figure 1B-D), Sch9p inactivation extended CLS, reduced O_2_**^-^** and lowered the fraction of budded cells detected in wild type cells cultured in 10% glucose YPD (Figure 3F-H). Since the CLS-shortening effects of 10% compared to 2% glucose are not related to acetic acid, we conclude that inactivation of Sch9p reduces O_2_**^-^** levels, enhances stationary phase G0/G1 arrest and extends CLS in 10% glucose medium by inhibiting glucose-dependent growth signaling, and not by causing resistance to acetic acid.

Wild type cells cultured in 2% glucose YPD medium also exhibited reduced levels of O_2_**^-^** compared to 2% glucose SC cultures (Figure S3; also compare “WT 2% glu” in Figure 3C with “WT 2% glu” in Figure 3G). This likely reflects a reduced amount of acetic acid in stationary phase YPD cultures compared to SC cultures, because the pH of stationary phase YPD medium is substantially higher than the pH of SC medium (Table 1). Furthermore, unlike in 2% glucose SC cultures (Figure 1B-C), in 2% glucose YPD cultures *sch9Δ* cells did not exhibit a longer CLS or reduced levels of O_2_**^-^** compared to wild type cells (Figure 3F-G). This suggests that in 2% glucose SC cultures, inactivation of *SCH9* extends CLS by inhibiting acetic acid induction of O_2_**^-^**.

### High glucose causes more frequent apoptotic elimination of dividing compared to non-dividing cells

**Figure 4.**
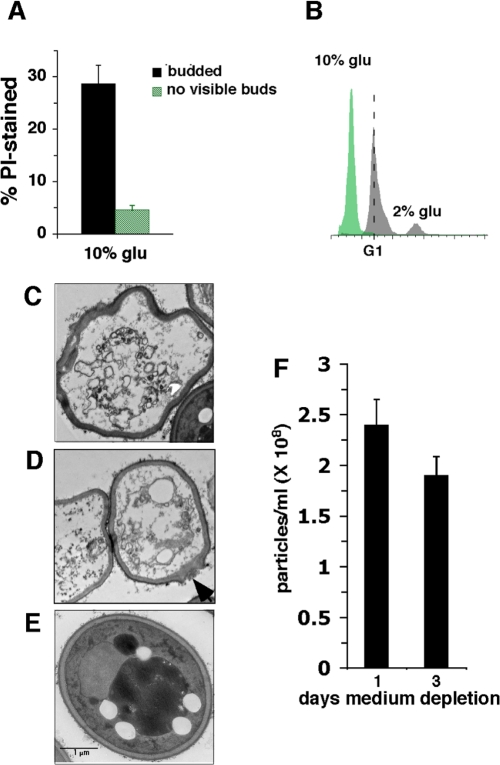
Effects of SC medium containing 10% or 2% glucose on cell death in stationary phase. (**A**) % wild type cells cultured in 10% glucose SC medium stained with propidium iodide (PI) at day 2 of medium depletion. (**B**) DNA content of wild type cells cultured in 10% or 2% glucose SC medium at day 2 of stationary phase. Dotted line marks cells with a G1 content of DNA. (**C-E**) Electron micrographs of stationary phase cells cultured in 2% glucose SC medium undergoing apoptosis (**C** and **D**) or not undergoing apoptosis (**E**). (**F**) Number of particles in 10% glucose SC cultures at day 1 and 3 of medium depletion.

The findings described in previous sections indicate that both glucose and acetic acid shorten CLS in concert with elevated levels of O_2_**^-^** and less efficient growth arrest of stationary phase cells in G0/G1. However, the reduced fraction of budded cells detected in 10% glucose compared to 2% glucose SC cultures (Figure 3E) is not consistent with a general relationship between enhanced growth signaling, increased O_2_**^-^** and less efficient G0/G1 arrest. Budding yeast cells die in stationary phase by an apoptosis-like mechanism [36,37]. The substantial increase in the fraction of stationary phase wild type cells with visible buds in 10% glucose YPD (Figure 3H) raised the possibility that the reduced fraction of budded cells in 10% glucose SC might be related to the very short CLS observed in these cultures and frequent apoptotic elimination of budded cells. Consistent with this possibility, PI staining of cells in 10% glucose SC stationary phase cultures revealed a 6-fold increase in the fraction of visibly budded cells that were dying compared to cells that did not have visible buds (Figure 4A). This is substantially larger than the ~2-fold increase in budded compared to unbudded cells that stain with PI in 2% glucose SC cultures (Figure S1).

Furthermore, at day 2 of medium depletion, cells in 10% glucose SC cultures were more frequently undergoing apoptosis compared to cells in 2% glucose SC indicated by increased apoptotic degradation of DNA. In fact almost all the cells in 10% glucose cultures harbored substantially less than the complete G1 complement of DNA required for continued viability (Figure 4B). Electron microscopic visualization of stationary phase cells cultured in 2% glucose YPD medium revealed that some cells exhibited fragmented nuclei indicative of apoptosis as well as an irregular cell shape indicating deterioration of the cell wall structure (Figure 4C and D). This contrasted with the appearance of intact nuclei and cell walls in non-apoptosing cells (Figure 4E). In some cases, disruption of the cell wall structure was detected at specific sites in apoptosing cells (Figure 4D; arrow) that may correspond to the location of a bud that broke off in cells undergoing apoptosis. A decline in numbers of cells in 10% glucose SC stationary phase cultures from day 1 to day 3 measured by counting particles (Figure 4F) confirmed that similar to mammalian cells, budding yeast cells undergoing apoptosis eventually are completely destroyed. We conclude that high glucose reduces the efficiency of G0/G1 arrest in stationary phase and preferentially kills dividing cells, and that the reduced number of cells with visible buds in 10% glucose SC cultures is caused by the specific and rapid apoptotic destruction of cells that failed to arrest growth in G0/G1.

### Superoxide anions inhibit stationary phase G0/G1 arrest

**Figure 5.**
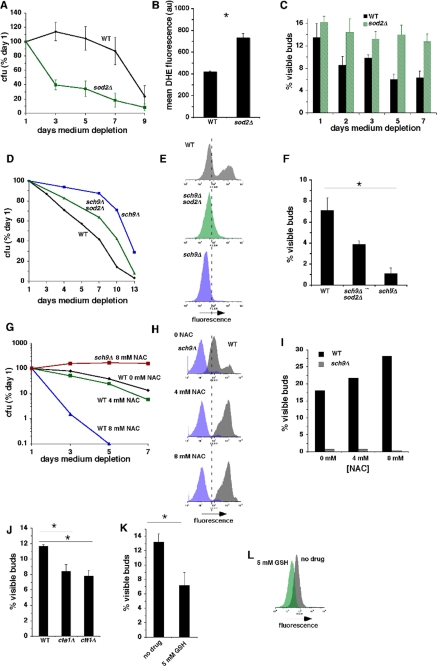
O_2_^-^ inhibit growth arrest of stationary phase cells in G0/G1 in parallel with a shorter CLS. (**A**) CLS of wild type and *sod2Δ* cells in 2% glucose SC medium. (**B**) Levels of O_2_**^-^** in wild type and *sod2Δ* cells detected by DHE fluorescence at day 3 of medium depletion. **C.** Fraction of cells with visible buds in wild type and *sod2Δ* cells at indicated times of medium depletion. (**D)** CLS of wild type, *sch9Δ* and *sch9Δ sod2Δ* cells. (**E**) Levels of O_2_**^-^** detected by DHE fluorescence in wild type, *sch9Δ* and *sch9Δ sod2Δ* cells at day 3 of medium depletion. (**F**) Fraction of cells with visible buds in wild type, *sch9Δ* and *sch9Δ sod2Δ* cells at day 3 of medium depletion. (**G**) Dose-dependent effects of NAC on CLS in wild type and *sch9Δ* cells. (**H** and **I**) Dose-dependent effects of NAC on O_2_**^-^** detected by DHE (**H**) and fraction of cells with visible buds (**I**) in wild type and *sch9Δ* cells at day 3 of medium depletion. (**J**) Fraction of cells with visible buds in wild type cells and the catalase mutants *cta1Δ* and *ctt1Δ* at day 3 of medium depletion. (**K**) Fraction of cells with visible buds at day 3 of medium depletion that were treated or not treated with the anti-oxidant GSH beginning at day 0. (**L**) Effect of GSH in wild type cells on levels of O_2_**^-^** indicated by DHE fluorescence at day 3 of medium depletion.

The inverse relationship between levels of O_2_**^-^** and the frequency with which cells arrest in G0/G1 when they enter stationary phase under a variety of experimental conditions (summarized in Table 2) suggests that O_2_**^-^** inhibits G0/G1 arrest. To test this hypothesis, we examined the effects of experimental manipulations that directly alter levels of O_2_**^-^** independently of changes in growth signaling pathways. *sod2Δ* cells exhibited a significantly shorter CLS (Figure 5A; [38]) accompanied by increased O_2_**^-^** (Figure 5B) and less efficient G0/G1 arrest (Figure 5C) in stationary phase. Sod2p expression is elevated in *sch9Δ* cells, and deletion of *SOD2* from *sch9Δ* cells partially suppresses their longevity phenotype ([4]; Figure 5D). *sch9Δ sod2Δ* double mutant cells also exhibited an intermediate level of O_2_**^-^** compared to wild type or *sch9Δ* cells (Figure 5E) accompanied by a stationary phase G0/G1 arrest that was intermediate between that of wild type and *sch9Δ* cells (Figure 5F). Thus, a quantitative relationship exists between levels of O_2_**^-^** and frequency of G0/G1 arrest in *sod2D, sch9D sod2Δ* and wild type cells.

**Table 2.** Summary of effects of experimental manipulations that impact CLS on superoxide anions and frequency of G0/G1 arrest in stationary phase

Experiment	Superoxide anions	G0/G1 arrest
**Longer CLS:**		
Deletion of *SCH9*	**↓**	**↑**
Deletion of *TOR1*	↓	↑
Deletion of *RAS2*	n.d.	↑
Caloric restriction	↓	↑
Buffering pH to 6.0	↓	↑
GSH	↓	↑
Deletion of *CTA1*	↓	↑
Deletion of *CTT1*	↓	↑
**Shorter CLS:**		
Deletion of *PDE2*	↑	↓
Deletion of *SNF1*	↑	↓
Deletion of *RIM15*	↑	↓
Deletion of *SIC1*	↑	↓
Deletion of *SOD2*	↑	↓
High glucose	↑	↓
N-acetylcysteine	↑	↓

We also asked whether treatment of cells with the antioxidant N-acetylcysteine (NAC) would extend CLS and increase the efficiency of stationary phase G0/G1 arrest in association with reduced levels of superoxide. Surprisingly, NAC shortened CLS in a dose-dependent fashion in wild type, but not *sch9Δ* cells (Figure 5G). The shorter CLS conferred by NAC in wild type cells occurred in concert with dose-dependent increases rather than decreases in levels of O_2_**^-^** (Figure 5H). Similar pro-oxidant effects of the antioxidants α-tocopherol and coenzyme Q10 were recently reported in budding yeast [39], and induction of O_2_**^-^** by NAC has been reported in mammalian cells as well [40]. Increased O_2_**^-^** in wild type cells exposed to NAC was accompanied by a parallel dose-dependent increase in the fraction of cells with visible buds (Figure 5I). NAC-induced increases in O_2_**^-^** and frequency of G0/G1 arrest in stationary phase were absent in *sch9Δ* cells (Figure 5H and I; “*sch9Δ*”). The absence of NAC effects in *sch9Δ* cells expressing elevated levels of Sod2p [4] suggests that in wild type cells, the effects of NAC are related to increased amounts of O_2_**^-^** and not to unrelated toxic effects of this compound. In contrast, cells in which the catalases Cta1p or Ctt1p had been inactivated, which exhibit reduced levels of O_2_**^-^** in stationary phase and a longer CLS [8], also exhibited fewer visible buds (Figure 5J). Similarly, cells treated with the antioxidant glutathione (GSH) also exhibited fewer visible buds (Figure 5K) in concert with reduced levels of O_2_**^-^** (Figure 5L). We conclude that O_2_**^-^** inhibits growth arrest of stationary phase cells in G0/G1.

## DISCUSSION

### Growth signaling and superoxide anions in the chronological aging model

**Figure 6.**
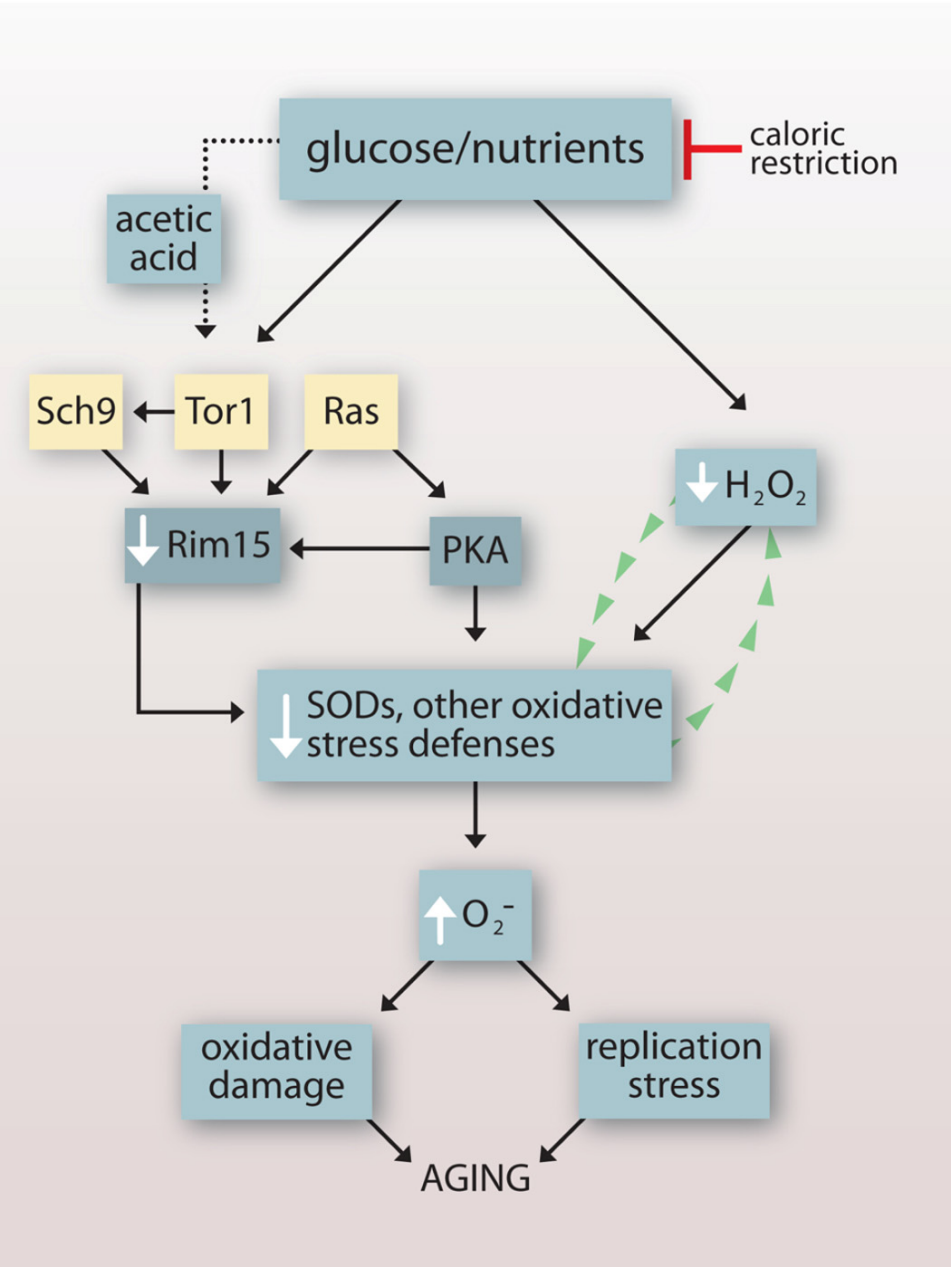
Impact of growth signaling pathways and caloric restriction on chronological lifespan in budding yeast. Glucose and other nutrients signal growth through conserved Sch9p-, Tor1p- and Ras-dependent pathways that inhibit Rim15p and its induction of oxidative stresses defenses, leading to elevated O_2_^-^ that cause oxidative damage and DNA replication stress. Acetic acid induces O_2_^-^ by activating the same pathways. Caloric restriction attenuates signaling through these pathways and also induces H_2_O_2_ that activates SODs and reduces levels of O_2_^-^ independently of Rim15p. In caloric restriction conditions, H_2_O_2_ that accumulates as a byproduct of increased SOD activity might stimulate SOD activity further by a self-amplifying mechanism.

Our findings reveal that under a variety of experimental conditions, an inverse relationship exists between budding yeast CLS and intracellular levels of O_2_**^-^** (summarized in Table 2) that points to O_2_**^-^** accumulating downstream of growth signaling as a primary cause of chronological aging. A role for growth signaling-induced O_2_**^-^** in chronological aging is consistent with earlier reports that CR extends CLS in part by downregulating Tor1p-, Ras2p- and Sch9p-dependent growth signaling pathways that inhibit the Rim15p kinase and its induction of oxidative stress defenses ([7]; Figure 6). Our findings also indicate that the Rim15p-independent extension of CLS by CR reported earlier [7] is related to the induction of H_2_O_2_ that reduces O_2_**^-^** (Figure 1G) by activating SODs [8] independently of Rim15p (Figure 6).

Burtner et al. [12] recently proposed that the primary cause of chronological aging in budding yeast is toxic effects of acetic acid that are not caused by oxidative stress, and that inactivation of Sch9 or Ras2 protect against acetic acid toxicity through unknown mechanisms rather than reduced growth signaling. Our data are consistent with a role for acetic acid toxicity as a determinant of CLS in 2% glucose medium. However, acetic acid causes O_2_**^-^** to accumulate in stationary phase cells, because buffering SC medium to a higher pH, which extends CLS, reduces levels of O_2_**^-^** [18]. O_2_**^-^** levels are similarly reduced in cells in YPD (Figure S3), which in addition to maintaining a higher medium pH (Table 1) exhibit a longer CLS (Figure 3; [13]). O_2_**^-^** accumulating in stationary phase cells is toxic, because experimental manipulations that directly elevate O_2_**^-^** levels (inactivation of Sod2p or exposure to NAC) shorten CLS (Figure 5).

Acetic acid [41] and/or intracellular acidification [42] induce the same TOR- and RAS-dependent growth signaling pathways induced by glucose, and the induction of O_2_**^-^** by acetic acid is likely a consequence of acetic acid-induced growth signaling. A role for RAS-dependent growth signaling in acetic acid toxicity is consistent with an earlier report that the enhanced stationary phase viability of *ras1Δ* and/or *ras2Δ* cells cultured in SC medium is absent in YPD cultures or in SC cultures buffered to a higher pH [43]. Our finding that CLS extension in *sch9Δ* compared to wild type cells cultured in 2% glucose SC (Figure 1B; [3]) is similarly absent when these cells are cultured in 2% glucose YPD, (Figure 3F), which also maintains a higher pH, suggests that acetic acid also triggers Sch9p-dependent growth signaling pathways. Therefore, the protective effects against acetic acid toxicity in unbuffered 2% glucose medium associated with inactivating Ras1p, Ras2p and Sch9p are likely due to downregulation of growth signaling by acetic acid and consequent upregulation of SODs and other oxidative stress defenses by Rim15p (Figure 6).

Burtner et al. also proposed that the effects of acetic acid on CLS are specific for this form of acid [12]. However, deletion of *RAS1* and *RAS2* also protects against acid stress induced by hydrochloric acid [43]. Furthermore, Ras2p-dependent growth signaling is triggered by the acidifying protonophore 2,4-dinitrophenol [42]. Low pH also induces AKT activity in human cells [44,45], and as we noted earlier [18], growth signaling by low pH that depends on RAS, AKT and other oncogenes underlies a number of pathological states in humans, including cancer. Thus, acetic acid toxicity in budding yeast corresponds to a conserved mitogen-like response to low pH, and not just acetic acid, that mimics the sustained activation of oncogenes in complex eukaryotes.

### Superoxide anions and effects of enhanced growth signaling by high glucose

In mammalian cell cultures, high levels of glucose that mimic the effects of hyperglycemia induce DNA damage, AKT-dependent growth signaling, increased O_2_**^-^** and senescence [31,34,35,46]. Each of these effects has been implicated in aging and age-related diseases linked to hyperglycemia and/or excess calorie intake, including cancer, diabetes and cardiovascular disease. High glucose also promotes aging in *C. elegans* in association with increased O_2_**^-^** [30]. The induction of elevated O_2_**^-^** and a shorter CLS by glucose signaling has been implicated in aging in the fission yeast *S. pombe* as well [47]. The increased levels of O_2_**^-^** (Figure 3C and G) and shorter CLS (Figure 3A and F) induced by 10% glucose in either SC or YPD medium establish budding yeast as an additional model for investigating the effects of elevated glucose on aging and age-related diseases. These effects are mediated in part by Sch9p-dependent signaling by glucose because in 10% glucose YPD cultures*, sch9Δ* cells exhibit a longer CLS and less O_2_**^-^** compared to wild type cells (Figure 3) in the absence of changes in pH compared to 2% glucose YPD cultures.

Changes in levels of acetic acid also do not play a role in the CLS-extending effects of increased H_2_O_2_ induced by CR [8]. The longer CLS in 2% compared to 10% glucose SC or YPD cultures in the absence of a change in medium pH reveals an additional mechanism by which CR extends CLS in budding yeast related to reduced growth signaling by glucose rather than acetic acid. CR is most often defined in yeast experiments as a decrease in the glucose content of medium below 2%. However, in their natural environment yeasts are likely exposed to higher concentrations of glucose and other sugars that trigger growth signaling. For example, the glucose and fructose content of grapes can exceed 13% [48] and the sugar content of overripe plantains approaches 27% [49]. Thus, culturing cells in 2% compared to 10% glucose medium can be considered a physiologically relevant form of CR that depends on reduced growth signaling by glucose rather than acetic acid. This form of CR is broadly relevant to CR in complex eukaryotes.

### Growth signaling, superoxide anions and DNA replication stress

The longstanding free radical theory of aging predicts that the pro-aging effects of O_2_**^-^** are caused by oxidative damage to macromolecules. However, the reduced levels of O_2_**^-^** and extended CLS produced by inactivation of catalases are accompanied by increased, rather than decreased oxidative damage [8]. Conversely, the shorter CLS detected in *sod2Δ* and other cells harboring defective oxidative stress defenses is not accompanied by general increases in oxidative damage to macromolecules [50,51] A similar disconnect between oxidative damage and longevity is observed in naked mole rats, which exhibit a ~10-fold longer lifespan compared to mice despite the presence of high levels of oxidative damage [52]. This suggests that the pro-aging effects of oxidative stress are not always a direct consequence of oxidative damage.

The inverse relationship between CLS and levels of O_2_**^-^** we detected under a variety of experimental conditions is accompanied by a similar inverse relationship between levels of O_2_**^-^** and frequency of G0/G1 arrest in stationary phase (Table 2). This points to an alternative, but not mutually exclusive possibility - that the age-promoting effects of O_2_**^-^** are related in part to inhibition by O_2_**^-^** of growth arrest of stationary phase cells in G0/G1, leading to more frequent growth arrest in S phase instead, where cells suffer replication stress. The inhibitory effects on G0/G1 arrest in stationary phase of experimental manipulations that more directly impact levels of O_2_**^-^** compared to alterations in growth signaling pathways (Figure 5) are consistent with this model.

Based on measurements of the fraction of stationary phase cells with visible buds, Madia et al. proposed that the effects on chronological aging related to replication stress are minor compared to other pro-aging factors [53]. In fact, the magnitude of inhibitory effects on G0/G1 arrest in stationary phase related to O_2_**^-^** is larger than suggested by counting cells with visible buds in stationary phase, for several reasons. First, cells die in stationary phase cultures via an apoptosis-like mechanism ([36]; Figure 4B-D) that eventually destroys cells (Figure 4F). The preferential death of cells that failed to arrest in G0/G1 (Figures. S1 and 4A) leads to underestimates of the fraction of these cells. Second, our data suggest that as the budding yeast cell wall deteriorates during apoptosis, buds break off of mother cells (Figure 4D), which would lead to additional underestimates of the fraction of cells with visible buds. Third, cells in early S phase with small buds are difficult to distinguish microscopically from unbudded cells that have truly arrested in G0/G1. Consequently, at least some of the dying cells that do not have visible buds in stationary phase in our experiments have not arrested in G0/G1.

According to a recent study, treatment of cells with low levels of hydroxyurea, which inhibits a protein essential for DNA replication (ribonucleotide reductase) shortens CLS by 20 - 27% [54]. Furthermore, increased apoptosis of stationary phase cells harboring a mutation in the replication stress protein Mec1 is suppressed by ectopic expression of the *RNR1* gene encoding ribonucleotide reductase [13]. Therefore, in principle, replication stress caused by reduced dNTP pools can substantially shorten CLS. Replication stress as a determinant of CLS is consistent with the observation that stationary phase cells that fail to arrest in G0/G1 die faster than unbudded cells (Figures S1;[18]). The rate at which dividing cells die in stationary phase is accelerated further when growth signaling and levels of O_2_**^-^** are enhanced by high glucose (Figure 4A), which also triggers DNA damage responses (Figure 3B). These findings suggest that the toxic effects of O_2_**^-^** in stationary phase cells are caused in part by DNA damage specifically in cells that failed to growth arrest in G0/G1.

A role for replication stress in chronological aging is also consistent with an earlier report by Allen et al. that non-quiescent stationary phase cells separated from denser quiescent cells by density gradients more frequently undergo apoptosis and exhibit elevated expression of genes encoding proteins that respond to replication stress [19]. It is not consistent with the results of a recent genetic screen that identified budding yeast deletion mutants that exhibit an extended CLS in the absence of more frequent stationary phase growth arrest in G0/G1 [55]. However, this screen also failed to identify the numerous deletion strains with inactivated growth signaling pathways, including *sch9Δ*, that were previously reported to have an extended CLS. In fact, in this recently published study, *ras2Δ* cells that earlier studies indicated are long-lived in the CLS model exhibited a substantially shorter CLS compared to wild type cells.

Replication stress as a determinant of CLS also is not consistent with the recent claim by Madia et al. [53] that the denser fraction of stationary phase cells, which according to Allen et al. [19] are quiescent and exhibit fewer signs of genome instability-promoting replication stress, paradoxically exhibit an elevated mutation frequency compared to “non-quiescent” cells [53]. We note that the experiments of Allen et al. employed YPD medium, which prolongs CLS compared to CLS in SC medium (Figure 3; [13]) and maintains a fraction of quiescent cells exhibiting a higher density for weeks [19]. In contrast, Madia et al. employed SC medium in their experiments. Close inspection of the data in Figure 2 of Madia et al. indicates that although a denser fraction of cells initially accumulated at day 1 of stationary phase in their experiments, unlike the experiments of Allen et al., this fraction declined and the fraction of less dense non-quiescent cells increased during the next several days of stationary phase. Furthermore, the number of stationary phase cells in S phase increased during this same period of time (Madia et al., Figure S1A). Although the fraction of budded cells in both “quiescent” and “non-quiescent fractions continues to decline with increasing time in stationary phase despite an overall increase in cells in S phase, this likely reflects the specific apoptotic destruction of budded cells. In fact, flow cytometry measurements by Madia et al. of the DNA content of “quiescent” and “non-quiescent” wild type cells clearly indicate that at the three and five day stationary phase time points they employed to measure mutation frequency in their experiments, most of the wild type cells they defined as “quiescent” that exhibited a higher mutation frequency also harbored significantly more DNA compared to “non-quiescent” wild type cells, and thus were more frequently in S phase (Figure S1B of Madia et al.; compare “Lower Fraction” (quiescent) histograms with “Upper Fraction” (non-quiescent) histograms at each time point). Thus, in contrast to the experiments of Allen et al., the initially denser cells Madia et al. refer to as “quiescent” do not remain quiescent for more than a few days, most likely due to increased growth signaling by the larger amounts of acetic acid accumulating in SC medium compared to the YPD medium employed in the experiments of Allen et al. In the absence of nutrients required for efficient DNA replication in stationary phase cultures, entry of these cells into S phase is a recipe for replication stress and mutations.

### Growth signaling, replication stress and aging in complex organisms

The induction of insulin/IGF-1-like growth signaling pathways that depend on RAS, AKT, mTOR and other oncogenic proteins has been implicated in aging and a number of age-related diseases in humans, including many for which hyperglycemia and/or excess calorie intact are risk factors. In addition to elevated levels of ROS, DNA replication stress has been implicated in some of these diseases as well. For example, sustained oncogenic signaling that leads to growth arrest in S phase has been implicated in the senescent state of preneoplastic cells [33]. Similarly, inappropriate activation of growth signaling pathways in tauopathies and other neurodegenerative disorders promotes unscheduled entry of postmitotic neurons into S phase [56], where these cells also likely undergo replication stress. Thus, as in budding yeast, growth signaling may impact aging in more complex organisms, including humans, by inducing replication stress, in addition to oxidative stress.

As in budding yeast, replication stress in mammalian cells may be related to O_2_**^-^** inhibition of quiescence. Consistent with this possibility, MnSOD-defective mouse cells driven into a non-dividing state by contact inhibition exhibit elevated levels of O_2_**^-^**, a higher fraction of S phase cells and increased apoptosis [40]. Furthermore, O_2_**^-^**induced by hyperglycemia [57] or by a metabolite of polychlorinated biphenyls that cause cancer [58] inhibit DNA replication. These findings have important implications for understanding aging and age-related diseases. For example, although DNA replication stress now is generally accepted as a factor that contributes to tumorigenesis downstream of oncogene activation, it is not considered to be an initiating event [59]. However, high glucose inhibits progression of endothelial cells through S phase [60], and as in our yeast experiments, also induces DNA damage in human mesothelial cells [34]. It is possible, therefore, that hyperglycemia and other factors can initiate tumorigenesis by inducing replication stress that leads to mutational activation of oncogenes.

This model does not explain all facets of aging, which is multifactorial and exceedingly complex. A compelling argument can be made, for example, that autophagy is in an important component of lifespan regulation in all eukaryotes [61]. Even here, however, our model may be relevant - in budding yeast, CLS extension by spermidine, which activates autophagy, is accompanied by reduced levels of O_2_**^-^** and more frequent arrest of stationary phase cells in G0/G1 [62]. Overall, our results provide a framework for investigating the role of growth signaling and oxidative stress in aging that accommodates recent evidence pointing to pro-aging factors other than oxidative damage. They also suggest a novel mechanism by which normal aging can be impacted by diet and other environmental factors.

## METHODS

Strains employed in these studies are listed in Table S1. To assess CLS 50 ml cultures were inoculated with 1% (v/v) of a fresh overnight culture in either SC or YPD. SC was supplemented as described [63]. In experiments that employed media that initially contained 10% glucose, control 2% glucose cultures also contained 8% sorbitol to maintain equivalent osmolarity. Deter-mination of chronological life span, fraction of budded cells, flow cytometry measurements of DNA content and measurements of dihydroethidium (DHE) and dihydrorhodamine 123 (DHR) were as described previously [8,13]. N-acetylcysteine (NAC) and glutathione (GSH) were dissolved in growth medium, filter sterilized and added to cultures from 100mM (NAC) or 250mM (GSH) stocks at the start of experiments. Propidium iodide (PI) was used to assess viability of cells by mixing a 2μl of cells with an equal volume of 1mM PI on a microscope slide and examining the slide with a fluorescence microscope equipped with a Texas Red filter. Non-fluorescent cells were scored as intact (live) and fluorescent cells were scored as dead. The total number of cells in cultures was determined by particle counts using a Petroff Hauser counting chamber.

Samples for transmission electron microscopy were prepared according to [64]. Briefly, cells cultured at 30°C in YPD medium for 3 to 5 days were harvested by gentle centrifugation, washed in phosphate buffered saline (PBS) (pH=7.2), resuspended in 2.5%(v/v) glutaraldehyde in PBS and fixed for 40 min at room temperature. Cells were further fixed by 2% freshly prepared potassium permanganate in water for 1 hour at room temperature. Fixed cells were dehydrated with 30%, 50%, 75%, 85%, 95%, and 100% ethanol. Cells were transitioned with propylene oxide, infiltrated in Spurr resin (Electron Microscopy Sciences, PA). Resin was polymerized at 65°C overnight in the oven. 60 nm ultrathin sections were cut with a diamond knife, stained with 2% uranyl acetate and lead citrate and examined using a Hitachi H-7000 electron microscope, equipped with a 4K × 4K cooled CCD digital camera (Gatan, Inc., CA).

Values presented in graphs that contain error bars represent means and standard deviations from three or more independent experiments. Other results are representative of at least three independent experiments. Statistical analyses were performed using Student's *t*-test. P < 0.05 was considered statistically significant.

## SUPPLEMENTARY FIGURES

Figure S1. Identification of dead or dying cells in stationary phase by staining with the membraneimpermeable dye propidium iodide.

Figure S2. Effect of inactivation of TOR1 and/or caloric restriction on CLS (**A**), levels of superoxide anions detected by DHE (**B)** and fraction of cells that fail to arrest in G0/G0 stationary phase (**C**).

Figure S3. Inactivation of Sic1 inhibits growth arrest of stationary phase in in G0/G1 in the CEN.PK background.

Table S1. Strains employed in this study
