# Supplementary figures and images for: Growth signaling promotes chronological aging in budding yeast by inducing superoxide anions that inhibit quiescence
Source: Aging (Albany NY). 2010 Oct 27;2(10):709–26. doi: 10.18632/aging.100215 (PMC2993800; doi:10.18632/aging.100215)

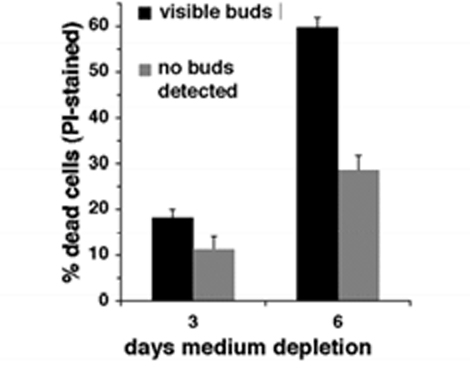

Supplement: Figure S1. — Identification of dead or dying cells in stationary phase by staining with the membraneimpermeable dye propidium iodide. [file aging-02-709-s001.tif]

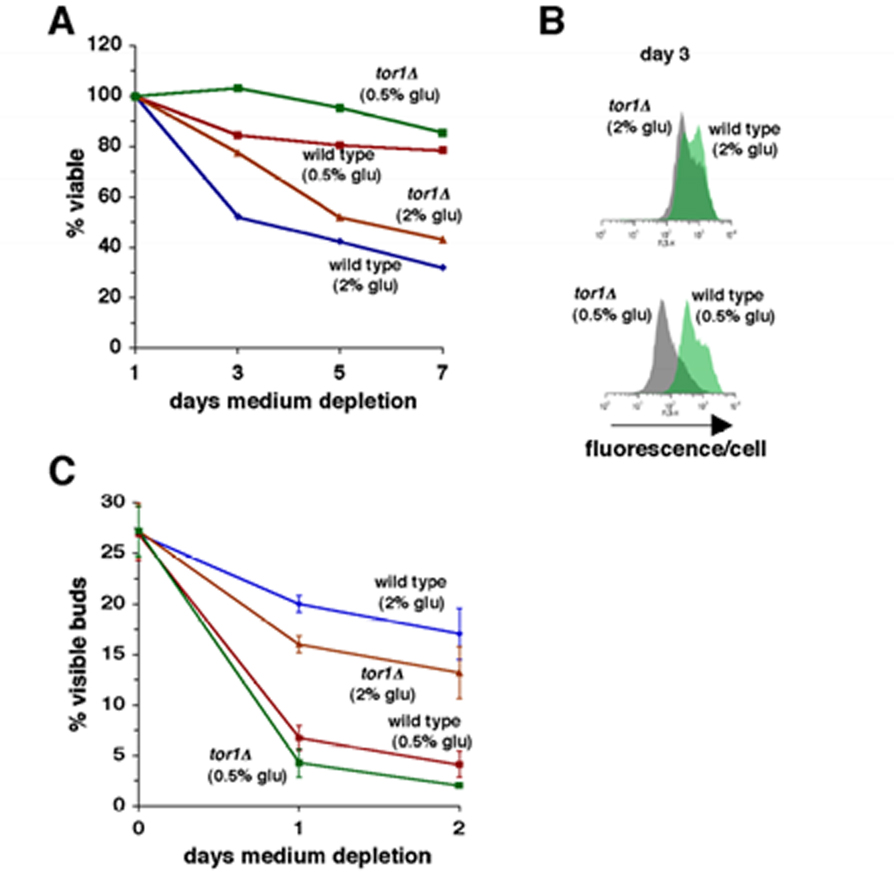

Supplement: Figure S2. — Effect of inactivation of TOR1 and/or caloric restriction on CLS (A), levels of superoxide anions detected by DHE (B) and fraction of cells that fail to arrest in G0/G0 stationary phase (C). [file aging-02-709-s002.tif]

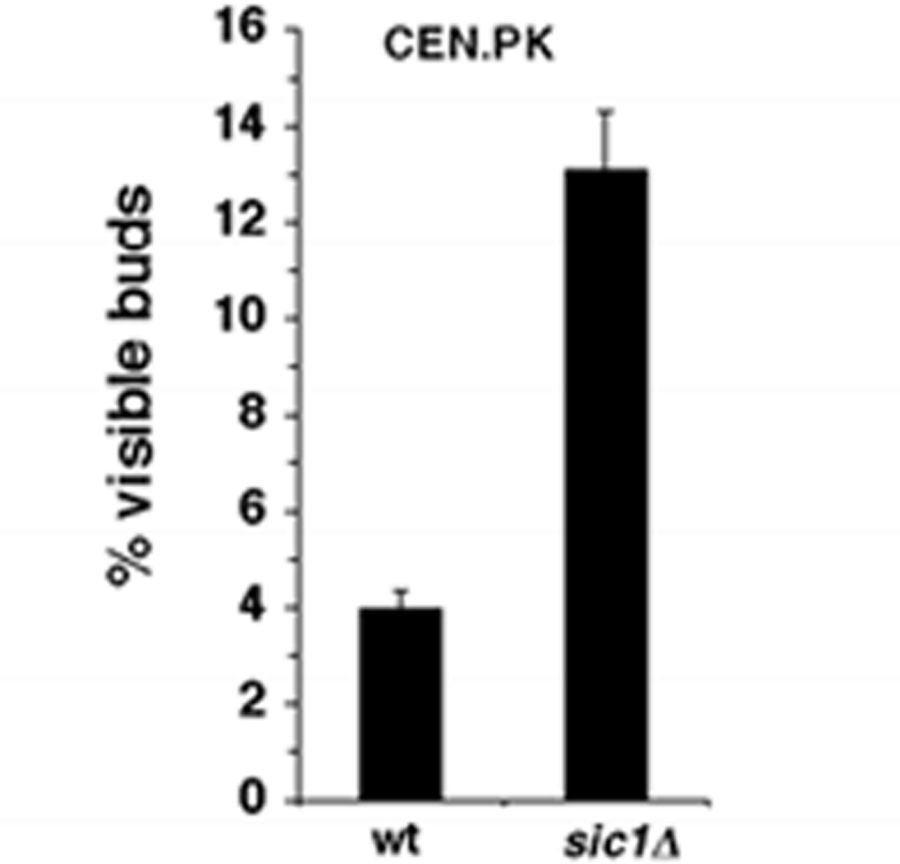

Supplement: Figure S3. — Inactivation of Sic1 inhibits growth arrest of stationary phase in in G0/G1 in the CEN.PK background. [file aging-02-709-s003.tif]

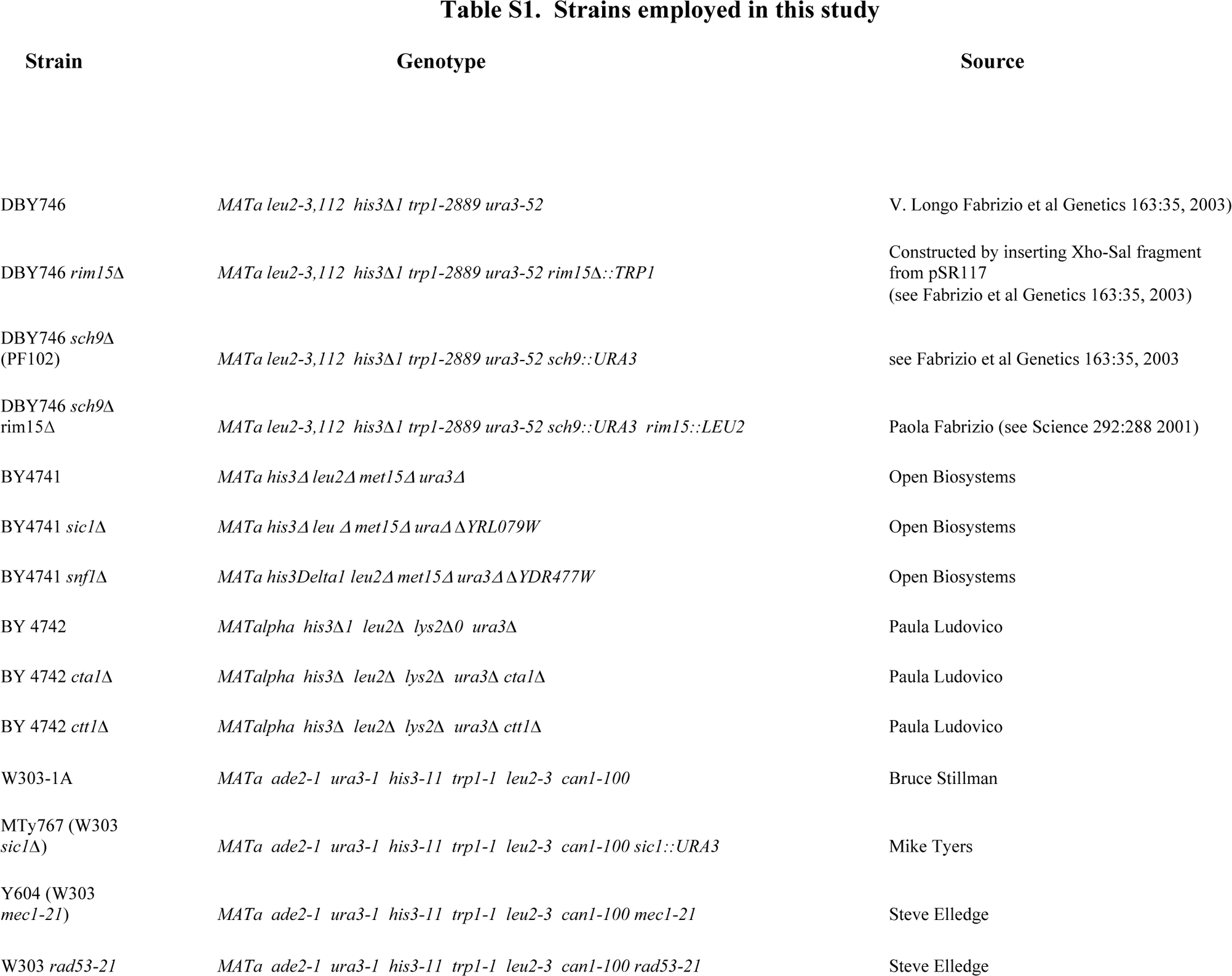

Supplement: Table S1. [file aging-02-709-s004.tif]
